# Case report: Thyroid metastasis from hepatocellular carcinoma: a rare case with diffuse solid occupancy and unusual imaging findings

**DOI:** 10.3389/fonc.2024.1360734

**Published:** 2024-07-10

**Authors:** Tinghua Feng, Menghua Xue, Miaoyu Sang, Rongrong Cui, Xiaofang Liu, Liping Liu

**Affiliations:** ^1^ Department of Ultrasound, Shanxi Bethune Hospital affiliated to Shanxi Medical University, Taiyuan, China; ^2^ Department of Endocrinology, The Third People’s Hospital of Datong, Datong, China; ^3^ Department of Ultrasound, First Hospital of Shanxi Medical University, Taiyuan, China; ^4^ Department of Interventional Ultrasound, The First Hospital of Shanxi Medical University, Taiyuan, China

**Keywords:** thyroid metastasis, hepatocellular carcinoma, diffuse thyroid lesion, echography, case report

## Abstract

**Background:**

Thyroid metastasis represents a rare occurrence, with commonly observed primary tumors originating from renal cell carcinoma, malignant neoplasms of the gastrointestinal tract, lungs, and breast. However, the metastasis of hepatocellular carcinoma to the thyroid gland remains infrequent. Previous investigations have consistently demonstrated an unfavorable prognosis for patients with malignancies that have metastasized to the thyroid. In this context, we present a noteworthy case of thyroid metastasis from hepatocellular carcinoma (HCC), characterized by a distinct ultrasonographic manifestation of diffuse thyroid lesion, deviating from the previously documented imaging presentations of thyroid metastases in HCC.

**Case presentation:**

A 62-year-old Chinese female patient was diagnosed with hepatocellular liver cancer in 2019, following which she underwent a radical hepatic resection. Pathological examination revealed HCC located in the right lobe (stage T3bN0M0 IIIB). No additional interventions were administered subsequent to the surgery. After a span of 15 months, the patient presented with dyspnea. Ultrasonographic findings showed diffuse solid infiltration within the thyroid gland, along with tumor thrombi in both internal jugular veins. Computed tomography (CT) scans demonstrated malignant thyroid lesions infiltrating the retropharyngeal space, prevertebral space, and esophageal wall. The subsequent pathology report from the puncture biopsy confirmed the malignant nature of the tumor, and immunohistochemical analysis definitively established its hepatic origin.

**Conclusions:**

Patients with a history of HCC should be subjected to long-term monitoring and habitual thyroid ultrasonography. Newly detected thyroid nodules in such patients should be immediately regarded suspect for potential metastatic disease. Even when a nodule doesn’t exhibit malignant characteristics on ultrasound, FNAB should be administered promptly to elucidate the pathological condition. Larger, swiftly multiplying thyroid masses should warrant an immediate CNB. The identification of thyroid metastases, particularly when coupled with peripheral tissue invasion, typically signifies a bleak prognosis.

## Introduction

In 1931, Willis proposed that the swift arterial circulation in the thyroid gland obstructed the adherence of malignant cells, while the gland’s oxygen saturation and iodine content impeded their growth. Over the past eight decades, the occurrence of thyroid metastases, contrary to Willis’ hypothesis, has shown variability across different studies (1). Metastatic involvement of the thyroid gland accounts for approximately 1.4% to 3% of all thyroid malignancies, but autopsy studies have reported incidence rates ranging from 1.9% to 24%, depending on the thoroughness of the examinations conducted (2). These figures underscore the fact that thyroid metastatic cancer frequently goes unnoticed and is often misdiagnosed in clinical settings. A recent review estimated that renal cell carcinoma accounted for 48% of malignant tumors metastasizing to the thyroid, followed by colorectal cancer at 10%, lung cancer at 8%, and breast cancer at 8% (3). HCC ranks among the most prevalent cancers worldwide and can manifest with both intrahepatic and extrahepatic metastases. The literature reports a thyroid metastasis rate of 2% in cases of HCC (4).This article presents a case study elucidating the metastasis of HCC to the thyroid gland, manifesting as an uncommon diffuse thyroid lesion. The case is subsequently deliberated in correlation with pertinent prior literature.

## Case presentation

**Figure 1:**
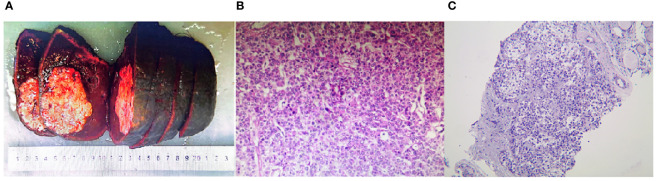
**(A)** The right hemihepatectomy specimen presents a perceivable mass in close proximity to the periosteum with a distinct boundary, embodying a hard texture and a grayish white hue. **(B)** Microscopic exploration of liver tissue, employing hematoxylin and eosin (H&E) staining, affirms the surgical pathology indicative of Hepatocellular Carcinoma (HCC). (H&E magnification of 100x). **(C)** Core Needle Biopsy (CNB) of the thyroid, post hematoxylin and eosin staining, corroborates the pathology of Hepatocellular Carcinoma metastasized to the thyroid. (HE magnification of 100x).

A female patient, aged 68, who had a history of hepatitis B in 2003, presented in August 2019 with epigastric discomfort primarily localized in the right upper abdomen and subxiphoid region, accompanied by a decrease in appetite. In November 2019, the patient returned with intermittent pain in the right upper abdomen that disrupted her sleep. Ultrasound and contrast-enhanced CT scans were performed, revealing the presence of a solid intrahepatic lesion, suggestive of malignancy. Physical examination revealed tenderness in the abdomen, specifically in the right upper region, without rebound pain. The liver and spleen were not palpable below the costal margin, but there was percussion pain in the liver region. The Murphy sign was negative, and no mobile turbidities or spider nevi were observed. Liver function analysis demonstrated an alkaline phosphatase level of 149.1 U/L and a glutamyl transferase level of 251.00 U/L. Following a radical hepatectomy for HCC in the right lobe (**Figures 1A, B**), the postoperative pathology results revealed hepatocellular HCC in the right lobe (stage IIIB, T3bN0M0), along with evidence of cancerous embolism in the right hepatic duct. Immunohistochemical analysis showed the following results: RCC (-), CD10 (-), PaX-8 (-), Hepar-1 (partially +), Glypican3 (+), CK7 (partially+), CK19 (partially+), Ki67 (40-50%). The patient received no other treatment after the surgery.

**Figure 2:**
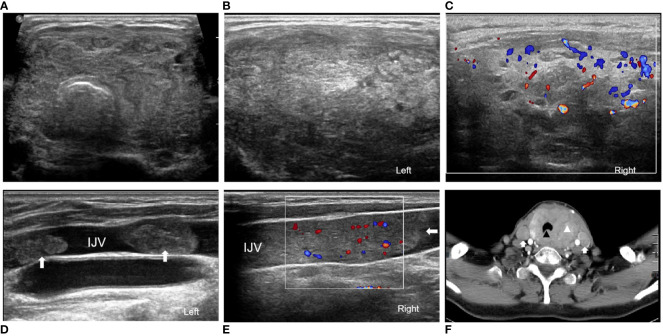
A case of metastatic HCC of the thyroid gland in a 68-year-old woman: **(A)** The transversal ultrasound image displays a thyroid gland enlarged, exhibiting a disproportionately large left lobe. **(B)** A sagittal ultrasonography displays widespread abnormal areas throughout the left section of the thyroid gland, devoid of any nodular entities. **(C)** Sagittal ultrasound depicts a multitude of abnormal areas in the right lobe of the thyroid gland, showcased with notable inconsistencies in parenchymal echogenicity and an elevated blood flow signal. **(D)** A sagittal ultrasound image reveals two solid tumor thrombi with identical echo features, both located within the left innominate vein. **(E)** A sagittal ultrasound image illustrates a single, solid tumor thrombus with consistent echo features in the right innominate vein, evidently showcasing increased blood flow within the thrombus. **(F)** The enhanced CT scan reveals an oversized thyroid gland with the inconsistent appearance of the thyroid gland, peripheral trachea (indicated with markers), the esophagus, and muscular engagement. Bilateral filling defects within the internal static veins are also discernible.

In March 2021, the patient sought medical help due to the onset of dyspnea. A solid, yet mobile mass was found in the left lobe of the thyroid gland during the physical examination, and it displayed vertical movability upon swallowing. Ultrasonographic imagery identified a dispersed solid mass within the thyroid region (**Figures 2A–C**) with poorly delineated boundaries with surrounding tissues. Tumor thrombi were discernible within the internal jugular veins bilaterally (**Figures 2D, E**). A neck CT scan indicated malignant thyroid lesions infiltrating the retropharyngeal space, prevertebral space, and the wall of the esophagus, with an accompanying tumor embolus visible within the bilateral internal jugular veins (**Figure 2F**). Hypermetabolic activity was detected in the thyroid area’s soft tissue mass, encircling the trachea and causing a reduced lumen via a PET/CT scan. An ultrasound-guided biopsy was carried out, with pathology suggesting malignancy (**Figure 1C**). Based on the patient’s medical history and immunohistochemical findings, the diagnosis suggested a poorly differentiated carcinoma, suspected to have originated in the liver. Immunohistochemical results were as follows: AE1/AE3(+), Vimentin(-), Hepatocyte (+), GPC -3(-), GS (-), AFP (-), HSP70(+), CD10(-), PAX-8(-), TTF-1(-), TG(-), CK19(-), Galectin-3(-), Mesothellal (-), CD56(-), Calcitonin(-), Syn (-), CGA (-), S-100(-), Ki67(40%). The patient and their family declined the proposed surgical intervention, citing considerable surgical risks as the reason. After a comprehensive evaluation of the patient’s overall health status, it was concluded that both radiotherapy and chemotherapy were unfeasible, given the patient’s capacity to tolerate them. Regrettably, the patient passed away following a two-month post-evaluation period.

Informed consent was obtained from the patient’s family for the publication of this article. The patient’s family regrets the missed opportunity for further treatment after the diagnosis of metastatic thyroid cancer. The patient’s relatives have no familial disease history or genetic predisposition.

## Discussion

HCC, a prevalent malignant neoplasm, has a distinct predisposition for vascular invasion, particularly the portal vein, facilitating the formation of intraventricular tumor thrombi leading to hemorrhagic distant metastases. Primary destinations of these metastases commonly include the lungs, skeletal structure, adrenal glands, and brain (5). Instances of intrathyroidal metastatic HCC are, however, strikingly infrequent. Intrathyroidal metastasis of HCC is rare, and a total of 10 cases, including the present case, have been reported in the PubMed database (8 cases) and Chinese database (1 case) (6–14) (**Table 1**).

**Table 1:** Summary of clinical features of previous cases of thyroid metastases from HCC.

Age/gender (References)	History of hepatitis	other thyroid diseases	thyroid function	Latency period (months)	grade and stage of primary tumor	treatment	Survival (months)	clinical manifestation
71/M (7)	nd	no	Euthyroidism	Synchronous	nd	Surgery and Chemotherapy	nd	Rapidly enlarging neck mass, difficulty swallowing
53/M (8)	yes	no	nd	7	T1N0M0	Surgery	8	Rapidly enlarging neck mass, dyspnea
73/M (9)	no	no	nd	22	T3N0M0	Surgery and Radiotherapy	Alive (13)	asymptomatic
62/F (10)	no	nodular goiter	Euthyroidism	17	T1NxMx, II	Surgery	Alive (36)	Mild dysphagia and neck enlargement
53/M (11)	yes	no	nd	9	nd	nd	20days	Neck swelling
63/M (12)	yes	no	Euthyroidism	24	nd	Radiotherapy	nd	Neck swelling, voice changes
41/M (13)	yes	no	nd	6	moderate-to-poorly	Surgery	Alive (22)	asymptomatic
62/M (14)	yes	Hashimoto's disease	nd	45	T1bN0M0	Surgery	6	Pain in the thyroid region
42/F (15)	nd	Hashimoto's disease	Euthyroidism	36	T2N0M0, II	Surgery	Alive (22)	Neck pain
present case/62/F	yes	no	Euthyroidism	15	T3bN0M0, IIIB	None	2	dyspnea

nd, no data.

Intrathyroidal metastatic HCC, owing to its astronomical rarity, leaves the age of onset and clinical features inadequately characterized. This study reveals an average onset age of 57.8 years during the metastasis of intrathyroidal metastatic HCC. Concurrent thyroid ailment was noted in three instances, splitting between nodular goiter and Hashimoto’s disease. Among these ten cases, a single one arose synchronously with HCC, while the remaining nine manifested heterochronously, affording an average latency of 20.1 months. A significantly higher male to female ratio (7:3) was discernible, reflecting the pervasive higher incidence and mortality rate of HCC in males (15).Patients showed salient clinical manifestations similar to primary thyroid neoplasms encompassing goiter, neck swelling, dysphagia, dysphonia, hoarseness, and coughing (16). These typical clinical symptoms could serve as pointers for detecting metastatic carcinoma in the thyroid gland, although such symptoms are infrequent thus often leading to the delayed diagnosis of thyroid metastases. Of the ten cases, our case was the sole one exhibiting diffuse involvement across the entire thyroid gland, while the other nine showcased focal nodules within the thyroid. Of these, six showed normal thyroid-liver function and the remaining four did not specify thyroid function status. Existing assertions suggest that extensive cancer cell infiltration could induce hypothyroidism. In spite of the diffuse lesion seen in this case, thyroid functionality didn’t appear to be compromised (**Table 1**).

Ultrasound remains the imaging modality of choice for thyroid disorders. Certain scholars have dichotomized secondary thyroid tumors into nodular (65%) and diffuse (35%) types based on their ultrasonographic traits (17, 18). The nodular type is typified by hypoechogenic intrathyroidal nodules, either solitary or multiple, exhibiting hypoechoic peripheries, ill-defined borders, devoid of hypoechoic halos or punctate calcifications, and varying degrees of vascular proliferation (4, 17). The case series presented herein consisted of nine nodular instances (**Table 2**). Five were solitary nodules, four flaunted multiple nodules, all presenting as hechoechoic and heterogeneous masses. In two cases the margins of the nodule were described as clear, and in only one case was the blood flow signal described as increased. Two cases were associated with ipsilateral lymph node metastasis, and one case revealed an attack on the thyroid cartilage. The diffuse type debuts as a diffuse hypoechoic lesion engulfing the entire thyroid gland, a nonspecific feature indistinguishable from thyroiditis or other diffuse thyroid disorders (18). The current case presents a classic diffuse type involving the entire thyroid gland with bilateral jugular intraventricular tumor thrombi and tracheoesophageal involvement. The invasion of surrounding tissues by tumor emboli alludes to a more advanced disease stage. Sonographic imaging of diffuse metastatic carcinoma of the thyroid commonly exhibits a diffusely enlarged thyroid gland with mixed parenchymal echogenicity and internal hypoechoic reticular lines, a pattern exclusive to diffuse lesions that sets them apart from other diffuse inflammatory thyroid disorders (19). However, the case of intrathyroidal metastatic HCC wasn’t part of the above study. Analysis of ultrasound images in this case occasionally revealed hyperechoic linear echoes, but the presentation deviated from common patterns depicted in previous literature. Blood flow signals were enhanced within the thyroid parenchyma and especially within the jugular vein tumor thrombus. The variability of these ultrasound findings underlines the necessity for sonographers to consider a range of differential diagnoses when evaluating thyroid lesions, assimilating the overall clinical picture, patient symptoms, and medical history.

**Table 2:** Reported cases of metastatic thyroid tumor from HCC.

Age/gender (References)	Thyroid ultrasound finding	Other Imaging	lesion diameter(cm)	Type of diagnosis	IHC
71/M (7)	Isolated heterogeneous solid mass in the **left lobe** of the thyroid gland	MRI showed a solitary thyroid nodule infiltrating the left sternocleidomastoid muscle and causing damage to the left thyroid cartilage plate.	5	CNB/HE	nd
53/M (8)	Multiple solid nodules in the left lobe of the thyroid gland with heterogeneous echogenicity	nd	nd	FNA/HE	TTF-1(-), CK7(-), CK20(-), TG(-), AFP(+)
73/M (9)	Isolated heterogeneous hypoechoic solid nodule in the **left lobe** of the thyroid gland	Whole-body screening using FDG-PET/CT revealed a low-density tumor in the left lobe of the thyroid gland that exhibited high fluorine-18 FDG uptake with a standardized maximum uptake value (SUVmax5.2)	nd	FNA	nd
62/F (10)	Multiple nodules in the **left lobe** of the thyroid gland	nd	7.5	FNA/HE	HepPar-1(+), CAM5.2(+), CD10(+), pCEA(+), TG(-), TTF-1(-)
53/M (11)	Multiple, solid masses of varying sizes in the **left lobe** of the thyroid gland are heterogeneous and hypoechoic without necrosis or calcification. The margins of the mass are well defined, and color Doppler examination does not reveal increased intratumoral or intranodal vascularity	nd	4	CNB	EMA(+), CK(+), CEA(-)
63/M (12)	Heterogeneous hypoechoic mass in the **left lobe** of the thyroid gland encroaching on the thyroid cartilage at the upper pole of the thyroid gland, with increased peripheral vascularization of the mass on Doppler examination	CT of the neck showed an irregular, inhomogeneous enhancing mass in the left lobe of the thyroid gland, invading the thyroid cartilage in the upper part of the left thyroid gland and compressing the airway; PET-CT showed focal strong uptake of the mass (SUVmax 4.6)	3.7	CNB/HE	GS(+), glypican-3(-), TTF-1(-)
41/M (13)	**Left lobe** thyroid lesion with cervical lymph nodes	18F-FDG PET/CT showed focal uptake in the left thyroid lobe, with localized activity enhancement (SUVmax 5.3)	nd	HE	Arginase1(+), HepPar-1(+), TG(-)
62/M (14)	The thyroid gland was enlarged, especially in the **left lobe**, and two hypophonic nodules were detected in the left lobe, fused to each other, with well-defined borders, a grid-like pattern within, and a strongly echogenic light spot visible within.	CT Enlargement of the left lobe of the thyroid gland with a hypodense nodular focus with indistinct margins, enhanced scan with marginal enhancement and a central hypodense area of less pronounced enhancement and more indistinct borders. No calcification was seen in the lesion. No enlarged lymph nodes were seen in the bilateral neck.	3.2	HE	CK (+) , CK19(+) , CK7(-) , Vimentin(-) , TTF-1(-) , TG (-) , Happar-1(-) , glypican 3(-) , Ki67(10%)
42/F (15)	Hypoechoic nodule in the left lobe of the thyroid gland (TI-RADS 4b), enlarged lymph node in the middle of the left neck	PET-CT showde hypodense nodule and metabolic increase in the left lobe of the thyroid gland(SUVmax 4.0)	nd	HE	Arginase1(+), HepPar-1(+), TTF-1(-)
present case/62/F	Diffuse echogenic changes in the thyroid parenchyma, poor demarcation of the thyroid peritoneum from the surrounding tissue, no nodular lesions	CT showed malignant occupying thyroid lesions involving the retropharyngeal space, prevertebral space, esophageal wall involvement, and bilateral internal jugular vein tumor thrombi. PET/CT scanning also suggested a hypermetabolic soft tissue mass in the thyroid region, which encircled the trachea with luminal stenosis	–	CNB/HE	AE1/AE3(+),Vimentin(-), Hepatocyte(+), GPC3(-), GS (-), AFP (-), HSP70(+), CD10(-), PAX8(-), TTF-1(-), TG (-), CK19(-), Galectin-3(-), Mesothellal(-), CD56(-), Calcitonin(-), Syn(-), CGA(-), S100(-), Ki67(40%+)

HE, histopathological examination; FNA, fine needle aspiration biopsy; CNB, core needle biopsy; IHC, immunohistochemistry; TTF, thyroid transcription factor; CK, cytokeratin; TG, thyroglobulin mAb; AFP, alpha fetoprotein; CD, Cluster of Differentiation; CAM, Cytokeratin; EMA,Epithelial membrane antigen; GPC, glypican; Hsp, Heat shock protein; PAX, paired box; Syn, synapsin; HepPar, Hepatocyte Paraffin; CEA, carcinoembryonic antigen; GS, glutamine synthetase; CGA,chromograninA; S100, Calcium Binding Protein; (+), positive immunostaining; (−), no immunostaining; nd, no data.

Computed Tomography (CT) and Magnetic Resonance Imaging (MRI) can provide valuable details on lesion enhancement and the neighboring anatomical structures. In the case series for this article (**Table 2**), two patients showed CT scans outlining a hypodense lesion with enhancement upon contrast injection, with one revealing invasion into the thyroid cartilage and compression of the airway. An MRI report indicated thyroid nodule presence with infiltration into the adjacent thyroid cartilage and sternocleidomastoid muscle in one patient. The CT in our case pointed towards tracheal and esophageal involvement and jugular vein tumor thrombosis. This aligns with existing literature on secondary malignant thyroid tumors’ presentation (20). Positron Emission Tomography-Computed Tomography (PET-CT) is a functional imaging technique that provides whole-body imaging. Its dominant advantage lies in detecting disease primary locus or monitoring tumor recurrent metastasis. In this paper, PET-CT scans implied thyroid nodules with elevated metabolism in four out of ten patients with thyroid-deriving metastases from HCC, with a maximum standardized uptake value (SUVmax) ranging between 4.0-5.2 (**Table 2**). This is generally concordant with previously reported outcomes from the literature (21). Hence, it has been suggested that PET-CT scans should be integrated into long-term follow-up programs for malignant tumor patients, which could aid in the early detection of thyroid metastatic foci. FDG-PET/CT is thus recommended for screening HCC patients for extrahepatic metastases or other malignancies before conducting surgical resection or liver transplantation and for managing extrahepatic metastases post-chemotherapy or radiotherapy (8). In patients with past or existing extrathyroidal malignancies, high suspicion should be maintained for possible thyroid metastasis when 18F-PET-CT scans exhibit goiter or diffuse infiltration of the thyroid gland.

Fine Needle Aspiration Biopsy (FNAB) is recognized as a pivotal diagnostic tool in thyroid pathology, encompassing thyroid metastasis, thereby forestalling redundant thyroidectomy in patients bearing an unfavorable prognosis (22). A comprehensive, multi-institutional examination of FNA revealed its efficacy in diagnosing thyroid malignancy in 87% of the participating subjects, of which 93% we’re definitively diagnosed with thyroid metastases (23). In recent studies, Core Needle Biopsy (CNB) has been fruitfully employed in diagnosing thyroid nodules. By procuring larger tissue specimen and a wealth of auxiliary histological data, CNB surpasses FNAB in diagnostic performance (24–26).CNB is the fundamental and preferred diagnostic instrument used specifically for sizeable and rapidly burgeoning thyroid masses such as Anaplastic Thyroid Carcinoma (ATC), or thyroid metastasis originating from thyroid lymphoma or other neoplastic entities (27, 28). As per current professional guidelines, CNB is advocated for thyroid nodules wherein repeated FNAB cytology proves to be inadequate, recurrent Bethesda class III cytology, and histological evaluation helps refine the preoperative diagnosis (e.g., suspicion of poorly differentiated or undifferentiated thyroid cancer, thyroid lymphoma, thyroid metastasis) (29). In patients harboring a clinical doubt of thyroid metastasis, CNB aids in curbing uncertain and erroneous findings, thereby thwarting needless and redundant diagnostic tests and procedures (30).

Immunohistochemical evaluations play a critical role in demarcating secondary thyroid malignancies from their primary counterparts. Namely, the detection of immunopositive staining for thyroglobulin and the thyroid transcription factor-1 (TTF-1) can indicate primary thyroid neoplasms (16). In contrast, secondary thyroid neoplasms typically do not display these markers; but instead, express unique indicators tied to their tissue of origin, such as alpha-fetoprotein (AFP) and hepatocytes. The presence of immunopositive staining for hepatocytes echoes a liver origin.

Surgery is pivotal in the management of secondary thyroid tumors. Among the 10 patients, 7 underwent surgery, with some receiving postoperative radiotherapy or chemotherapy. Unfortunately, our documented cases lacked access to therapeutic interventions. The current treatment options encompass thyroidectomy, lymph node dissection, and adjuvant therapy (31).

Thyroidectomy outperforms radiation or chemotherapy. A Mayo Clinic study revealed a median survival of 20 months for metastatic patients, with 41 out of 97 undergoing thyroidectomy.

Notably, no complications or deaths were reported. Patients who underwent thyroidectomy or received adjuvant therapy had a median survival of 30 months, compared to 12 months for those without thyroid surgery (32). Surgical treatment is feasible when metastases are confined to the thyroid gland.

As therapeutic measures for advanced disorders are continually evolving, new methodologies are consistently being developed. Regarding prevailing HCC, anti-angiogenesis modulations have claimed a pivotal role in the treatment plan. Examples might include angiogenesis inhibitors, such as sorafenib and lenvatinib, which halt tumor angiogenesis and thus reduce the risk of vascular intrusion and distant metastasis (33).. Recent analyses suggest that immune checkpoint inhibitors like pembrolizumab and nivolumab have shown effectiveness in patients with advanced HCC who have shown progression on sorafenib therapy or have an intolerance to it (34). These pharmaceuticals target vascular endothelial growth factor (VEGF) receptors and other angiogenesis-oriented pathways integral to the proliferation and metastatic dissemination of tumors (35). Immunotherapeutic avenues might wield more potential in treating metastatic HCC compared to conventional palliative surgical interventions. To conclude, when contending with patients suffering from advanced metastatic malignancies, a tailor-made treatment approach involving multi-disciplinary coordination should be adopted, taking into consideration the inherent properties of the primary malignancy and metastatic sites, in tandem with the patient’s surgical tolerance and projected lifespan.

This paper presents a rare case of metastatic thyroid carcinoma (HCC) with a unique ultrasound presentation that differs from previous reports. However, this study has its limitations. First, the study only includes one case and a review of relevant literature, which means it has a relatively small sample size and the conclusions may not be generalizable to the entire population. Further case studies are needed to validate and better understand the characteristics of this metastasis. Second, there are individual differences in patient condition and response to treatment, which may affect the applicability of the findings to all patients with similar conditions. In addition, the case report lacks an in-depth analysis of the impact of treatment options on prognosis, focusing mainly on descriptive aspects. The aim of this article is to improve the diagnosis and management of metastatic thyroid cancer (HCC). To validate and extend these findings, further studies will be necessary in the future. These studies could also discuss the molecular mechanisms of intra-thyroidal metastatic HCC, identify new therapeutic targets, and optimize therapeutic strategies to improve patient prognosis.

## Conclusions

To summarize, patients with a history of HCC should be subjected to long-term monitoring and habitual thyroid ultrasonography. Newly detected thyroid nodules in such patients should be immediately regarded as suspect for potential metastatic disease. Even when a nodule doesn’t exhibit malignant characteristics on ultrasound, FNAB should be administered promptly to clarify the pathological condition. Larger, swiftly multiplying thyroid masses should warrant immediate consideration for a CNB. The identification of thyroid metastases, particularly when coupled with peripheral tissue invasion, typically signifies a bleak prognosis. Early detection and accurate diagnosis are crucial in the treatment and management of patients. For advanced patients, individualized treatment strategies based on tumor characteristics, degree of metastasis, and patient-specific factors are necessary.

## Data availability statement

The original contributions presented in the study are included in the article/**Supplementary Material**. Further inquiries can be directed to the corresponding author.

## Ethics statement

The studies involving humans were approved by Ethics Committee of Shanxi Bethune Hospital. The studies were conducted in accordance with the local legislation and institutional requirements. The participants provided their written informed consent to participate in this study. Written informed consent was obtained from the individual(s) for the publication of any potentially identifiable images or data included in this article.

## Author contributions

TF: Writing – original draft, Conceptualization, Data curation, Formal analysis. MX: Writing – review & editing, Investigation, Visualization, Supervision. MS: Writing – review & editing, Investigation. RC: Writing – review & editing. XL: Writing – review & editing. LL: Writing – review & editing.
