# Supplementary figures and images for: Case report: Thyroid metastasis from hepatocellular carcinoma: a rare case with diffuse solid occupancy and unusual imaging findings
Source: Front Oncol. 2024 Jul 10;14:1360734. doi: 10.3389/fonc.2024.1360734 (PMC11266162; doi:10.3389/fonc.2024.1360734)

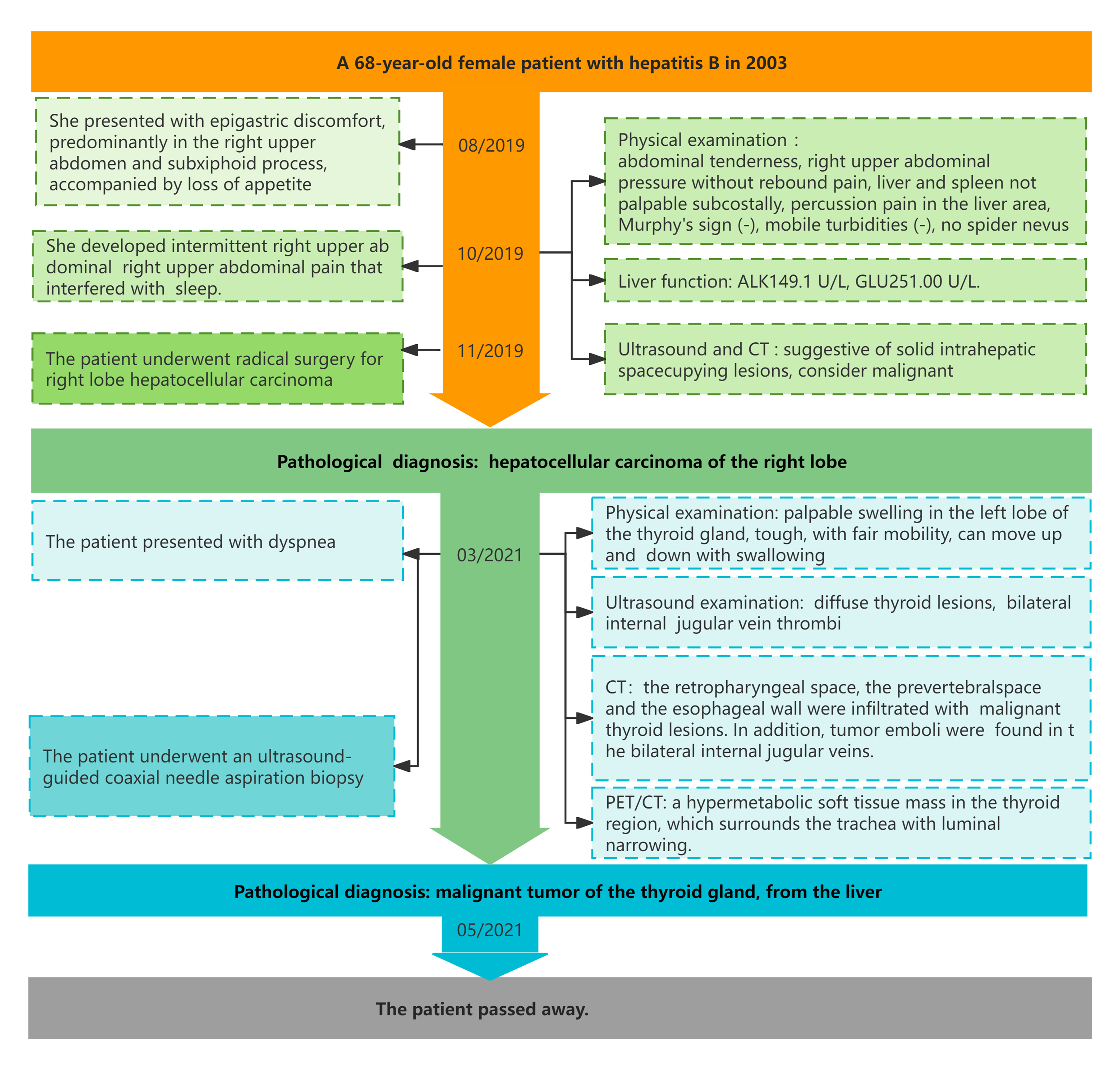

Supplement: Supplementary file 1 [file Image_1.tif]
